# MiRNA-disease association prediction via hypergraph learning based on high-dimensionality features

**DOI:** 10.1186/s12911-020-01320-w

**Published:** 2021-04-20

**Authors:** Yu-Tian Wang, Qing-Wen Wu, Zhen Gao, Jian-Cheng Ni, Chun-Hou Zheng

**Affiliations:** 1 School of Software, Qufu Normal University, Qufu, China; 2 School of Computer Science and Technology, Anhui University, Hefei, China; 3 College of Mathematics and System Science, Xinjiang University, Urumqi, China

**Keywords:** MicroRNA, Disease, MiRNA-disease association, K-nearest-neighbor, Hypergraph learning

## Abstract

**Background:**

MicroRNAs (miRNAs) have been confirmed to have close relationship with various human complex diseases. The identification of disease-related miRNAs provides great insights into the underlying pathogenesis of diseases. However, it is still a big challenge to identify which miRNAs are related to diseases. As experimental methods are in general expensive and time‐consuming, it is important to develop efficient computational models to discover potential miRNA-disease associations.

**Methods:**

This study presents a novel prediction method called HFHLMDA, which is based on high-dimensionality features and hypergraph learning, to reveal the association between diseases and miRNAs. Firstly, the miRNA functional similarity and the disease semantic similarity are integrated to form an informative high-dimensionality feature vector. Then, a hypergraph is constructed by the K-Nearest-Neighbor (KNN) method, in which each miRNA-disease pair and its *k* most relevant neighbors are linked as one hyperedge to represent the complex relationships among miRNA-disease pairs. Finally, the hypergraph learning model is designed to learn the projection matrix which is used to calculate uncertain miRNA-disease association score.

**Result:**

Compared with four state-of-the-art computational models, HFHLMDA achieved best results of 92.09% and 91.87% in leave-one-out cross validation and fivefold cross validation, respectively. Moreover, in case studies on Esophageal neoplasms, Hepatocellular Carcinoma, Breast Neoplasms, 90%, 98%, and 96% of the top 50 predictions have been manually confirmed by previous experimental studies.

**Conclusion:**

MiRNAs have complex connections with many human diseases. In this study, we proposed a novel computational model to predict the underlying miRNA-disease associations. All results show that the proposed method is effective for miRNA–disease association predication.

## Background

MicroRNAs (miRNAs) are endogenous non-coding single-stranded RNA molecules that play important roles in eukaryotic gene expression through posttranscriptional regulation [1–3]. Functional studies indicate that miRNA plays a significant role in manifold biological processes, such as cell proliferation, stem cell maintenance, immune responses and so on [4–6]. Dysregulation of miRNA expression and function is reported in various diseases including cancer, metabolic disorders as well as neurological disorders [7]. Therefore, identifying disease-related miRNAs is important to treat, diagnose, and prevent human complex diseases [8, 9].

Generally, researchers use biological experimental methods such as quantitative reverse transcription, microarray analysis, or deep sequencing of small RNAs to explore miRNAs that are differentially expressed in a disease state. For example, Pan et al. used microarray analysis and found that miR-130a-3p, miR-424-5p, miR-574-5p, and miR-146a presented significant difference between tuberculous meningitis and healthy controls [10]. However, experimental identification of disease-related miRNAs by existing techniques is expensive and time-consuming. So, based on vast amount of biological data about miRNAs, researchers have developed computational methods for predicting miRNA-disease associations [11–21], which can select most promising miRNAs for further analysis and hence decrease the number of the experiments.

For predicting disease-related miRNAs, many methods are based on a credible assumption that functionally similar miRNAs tend to have associations with phenotypically similar diseases and vice versa. Xiao et al. proposed a method called GRNMF, which based on graph regularized non-negative matrix factorization from the similarity and association perspective of miRNAs and diseases to discover potential associations [22]. Liu et al. proposed the method for predicting miRNA–disease associations by performing random walks on heterogeneous omics data [23]. You et al. presented the prediction model of PBMDA by constructing a heterogeneous graph consisting of three interlinked sub-graphs, and performing a depth-first search algorithm on the heterogeneous network to infer disease-related miRNAs [24]. PBMDA integrated different types of heterogeneous biological datasets, so it can be applied to the new diseases/miRNAs without known associated miRNAs/diseases. Subsequently, Chen et al. proposed a novel method based on Hybrid Approach for MiRNA-Disease Association prediction (HAMDA) [25]. They considered network structure, information propagation, and node attribution, and used the hybrid graph-based recommendation algorithm to uncover disease-related miRNAs. In addition, Chen et al. devised a computational approach by Graphlet Interaction to predict disease-related miRNAs (GIMDA) [26]. In this method, graphlet interaction was utilized to analyze the complex relationships between two nodes in a graph. However, HAMDA and GIMDA are not applicable to predicting a new association between a new miRNA and a new disease. Furthermore, Chen et al. developed a method of Graph Regression for MiRNA-Disease Association prediction (GRMDA) [27]. The graph regression was synchronously performed in three latent spaces, by using Singular Value Decomposition (SVD) and Partial Least-Squares (PLS) to extract important related attributes and filter the noise. But it is difficulties to choosing parameters in SVD and PLS. Lately, Jiang et al. implemented a improved collaborative filtering-based method to infer miRNA-disease associations (ICFMDA) [28]. They improved collaborative filtering algorithm by combining the similarity matrices, and defined significance SIG between pairs of diseases or miRNAs to predict disease-related miRNAs even new diseases without known association.

In addition, several computational models used machine learning to uncover the association between miRNAs and diseases. Xu et al. introduced an approach based on the miRNA target–dysregulated network (MTDN) to prioritize novel disease miRNAs [29]. They applied Support vector machine classifier to miRNAs in the MTDN. However, negative samples required by the classifier are difficult to obtain. To overcome this limitation, Chen et al. introduced a semi-supervised method named RLSMDA [30]. It is developed under the framework of regularized least squares and can predict new miRNAs for diseases which do not have any known related miRNAs. Similarly, Luo et al. developed another semi-supervised method named KRLSM based on Kronecker regularized least squares [31]. KRLSM integrated different omics data, combined the disease and miRNA space, and used the semi-supervised classifier of regularized least squares to predict disease-related miRNAs. However, this approach involves multiple parameters and establishing the optimal parameter values remains a challenging problem. Chen et al. designed a method based on restricted Boltzmann machine for predicting miRNA-disease associations [32]. This approach can also predict association types of miRNA-disease pairs, but can not applicable to a new disease with no known associated miRNAs. Furthermore, Chen et al. developed an effective method called HGIMDA [33]. HGIMDA calculated the disease-miRNA association possibility by investigating all the 3-length paths in the constructed heterogeneous graph. Recently, Chen et al. utilized Extreme Gradient Boosting Machine to uncover disease-related miRNAs and named EGBMMDA [34]. In this method, based on statistical measures, graph theoretical, and matrix factorization, they constructed an informative feature vector for each miRNA-disease pair and used a decision tree model to predict disease-related miRNAs.

Although existing methods have made great contributions to uncover disease-related miRNAs, there are still some limitations that could be improved. For example, many methods are difficult to extract the deep feature representation of the multiple kinds of data. In this study, we propose a novel prediction method via hypergraph learning based on high-dimensionality features and refer to it as HFHLMDA. Hypergraph learning, which can capture the high-order relationships of samples, has been widely used in clustering, classification and information retrieval tasks. In a hypergraph, an edge connects more than two vertices, thus it can well encode the relationship among more than two vertices. We construct high-dimensionality feature vectors for all the miRNA-disease pairs, and utilize K-Nearest-Neighbor (KNN) method to form a hypergraph to predict potential miRNA-disease association. To demonstrate the effectiveness of our method, we apply Leave-one-out cross validation (LOOCV) and fivefold cross validation to measure the prediction performance. We compare our method with four state‐of‐the‐art methods and the results indicate that our method can achieve better performance. In addition, case studies of three common diseases are implemented to further verify the reliability and robustness of HFHLMDA.

## Methods

### Human MiRNA-disease associations network

The human miRNA-disease associations used in this work come from the HMDDv2.0 [35], which contains 5430 experimentally associations between 495 miRNAs and 383 diseases. Technically, we use an adjacency matrix *A* with 495 (*nm*) rows and 383 (*nd*) columns to clearly describe the relation of each miRNA-disease pairs. The element *A*(*m*(i), *d*(*j*)) is equal to 1 if miRNA *m*(*i*) is verified to be associated with disease *d*(*j*), and 0 otherwise. Finally, 5430 entries of matrix *A* are assigned 1, the rest ones are assigned 0. Our goal is to confirm the uncertain associations between miRNAs and diseases.

### MiRNA similarity matrix

Wang et al. developed a method named MISIM for calculating the function similarity scores of miRNA [36]. Here, we directly downloaded the miRNA functional similarity scores from http://www.cuilab.cn/files/images/cuilab/misim.zip. Then, an adjacency matrix *SM* with 495 rows and 495 columns is built to denote the similarity of miRNAs, in which the larger the *SM*(*m*(*i*), *m*(*j*)) is, the more similar *m*(*i*) and *m*(*j*) are.

However, *SM* has the problem of sparsity. Sparse matrix is difficult to provide more effective information, which will seriously affect the prediction performance of the computational model. So we calculate the Gaussian interaction profile kernel similarity of miRNAs [37]. Specifically, a binary vector *BV*(*m*(*i*)), i.e. the *i*th row of matrix *A*, is recorded as the interaction profiles of miRNA *m*(*i*) for representing the associations between *m*(*i*) itself and each disease. All known miRNA-disease associations in matrix *A* will be used to calculate similarity, two miRNAs would likely have greater similarities if they share more disease associations. Thus, the Gaussian interaction profile kernel similarity *GKM*(*m*(*i*), *m*(*j*)) of miRNA *m*(*i*) and miRNA *m*(*j*) is defined as

1 $$GKM\left( {m\left( i \right),m\left( j \right)} \right) \, = {\exp}( - \gamma_{m} ||BV\left( {m\left( i \right)} \right) \, - BV\left( {m\left( j \right)} \right)||^{{2}} )$$

where *γ*_*m*_ is a parameter used to control the kernel bandwidth, which is set as

2 $$\gamma_{m} = \frac{1}{{\frac{1}{nm}\mathop \sum \nolimits_{i = 1}^{nm} ||BV\left( {m\left( i \right)} \right)||^{2} }}$$

By integrating *SM* and *GKM*, a new complete miRNA similarity matrix *SM* can be obtained as

3 $$SM\left( {m\left( i \right),m\left( j \right)} \right) \, = \left\{ {\begin{array}{*{20}l} {GKM\left( {m\left( i \right),{ }m\left( j \right)} \right) } \hfill & {if SM\left( {m\left( i \right),{ }m\left( j \right)} \right) = 0} \hfill \\ {\frac{{SM\left( {m\left( i \right),{ }m\left( j \right)} \right) + GKM\left( {m\left( i \right),{ }m\left( j \right)} \right)}}{2}} \hfill & {otherwise} \hfill \\ \end{array} } \right.$$

### Disease similarity matrix

The association between different diseases can be represented by a directed acyclic graph (DAG), which consists of some nodes and links. Each node represents a disease while a link represents the association of two diseases. For a given disease *D*, DAG = (*D*, *T*_*D*_, *E*_*D*_), where *T*_*D*_ represents its ancestor nodes and itself while *E*_*D*_ is the set of corresponding edges. The contribution values of disease *d*(*t*) to the semantic value of disease *d*(*i*) can be calculated as follows:

4 $$D_{d\left( i \right)} (d(t)) \, = - log\left( {\frac{{the\, number\, of\, DAGs\, including d\left( t \right){ }}}{the\, number \,of\, diseases}} \right)$$

5 $$DV\left( {d\left( i \right)} \right) = \mathop \sum \limits_{{d\left( t \right) \in D\left( {d\left( i \right)} \right) }} D_{d\left( i \right)} \left( {d\left( t \right)} \right)$$

where *D*(*d*(*i*)) is the node set in DAG(*d*(*i*)) including node *d*(*i*) itself. Therefore, the semantic similarity between disease *d*(*i*) and *d*(*j*) can be defined as follows:

6 $$SD\left( {d\left( i \right),d\left( j \right)} \right) = \frac{{\mathop \sum \nolimits_{{d\left( t \right) \in D\left( {d\left( i \right)} \right) \cap D\left( {d\left( j \right)} \right)}} \left( {D_{d\left( i \right)} \left( {d\left( t \right)} \right) + D_{d\left( j \right)} \left( {d(t} \right))} \right)}}{{DV\left( {d\left( i \right)} \right) + DV\left( {d\left( j \right)} \right)}}$$

Similarly, we also calculate the Gaussian interaction profile kernel similarity *GKD* for diseases by the follow formulas

7 $$GKD\left( {d\left( i \right),d\left( j \right)} \right) \, = {\exp}( - \gamma_{d} ||BV\left( {d\left( i \right)} \right) \, - BV\left( {d\left( j \right)} \right)||^{{2}} )$$

8 $$\gamma_{d} = \frac{1}{{\frac{1}{nd}\mathop \sum \nolimits_{i = 1}^{nd} ||BV\left( {d\left( i \right)} \right)||^{2} }}$$

where *BV*(*d*(*i*)) and *BV*(*d*(*j*)) denote the *i*th column and the *j*-th column of *A*. At last, the disease similarity matrix *SD* is obtained by

9 $$SD\left( {d\left( i \right),d\left( j \right)} \right) \, = \left\{ {\begin{array}{*{20}l} {GKD\left( {d\left( i \right),{ }d\left( j \right)} \right)} \hfill & {if\,SD\left( {d\left( i \right),{ }d\left( j \right)} \right) = 0} \hfill \\ {\frac{{GKD\left( {d\left( i \right),{ }d\left( j \right)} \right) + SD\left( {d\left( i \right),{ }d\left( j \right)} \right)}}{2}} \hfill & {otherwise} \hfill \\ \end{array} } \right.$$

### HFHLMDA

**Fig. 1:**
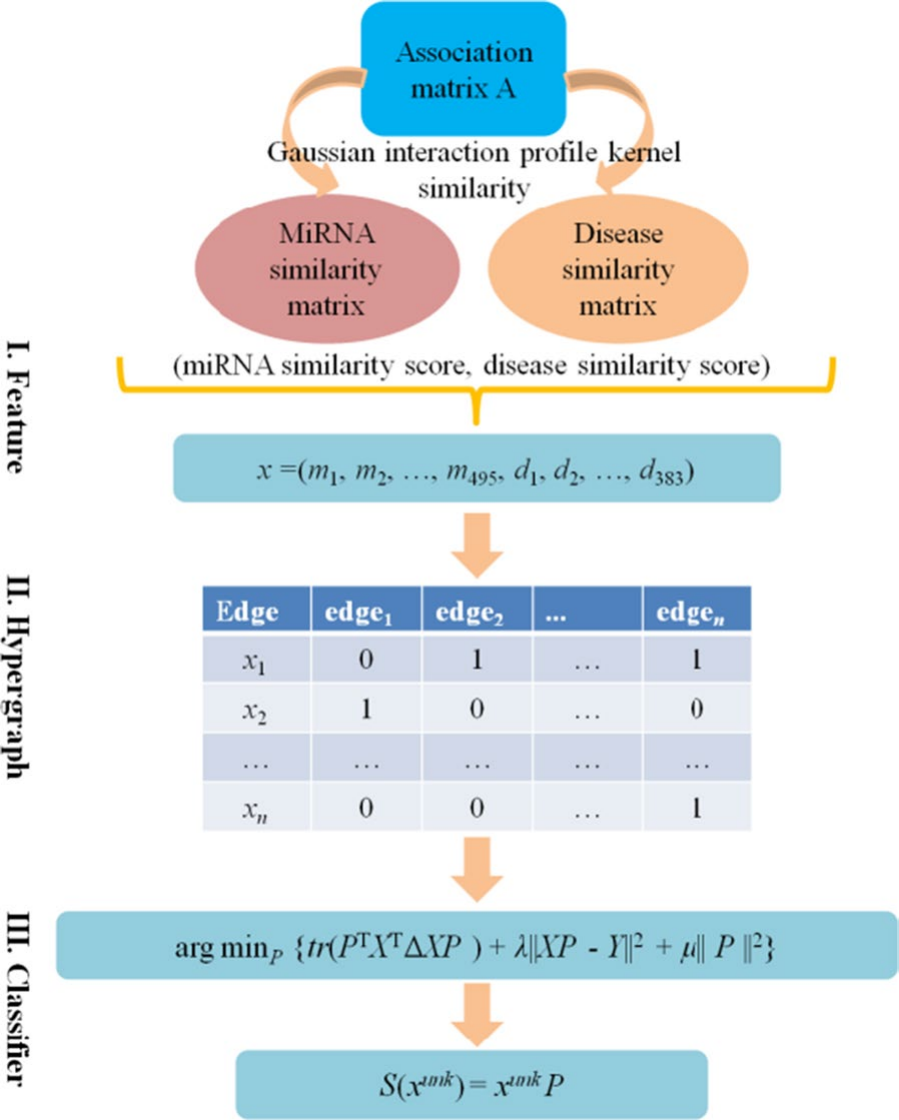
Flowchart of potential miRNA–disease association prediction based on HFHLMDA

The HFHLMDA model can be separated into three steps (see Fig. 1). First, feature factor construction, in which a feature factor *x* for each miRNA-disease pair consisting of corresponding rows of *SM* and *SD*. Second, hypergraph construction, where a hypergraph *G* is constructed to formulate the relationship between these feature vectors. Third, hypergraph learning, to learn the projection matrix *P*, which map the original feature *x* to the relevance score *S* = *x*^.^*P*, and thus it can be used to predict the association for the unknown miRNA-disease pair *x*^*unk*^.

### Feature factor construction

According to the biological observation that miRNAs with more functional similarity tend to be more associated with similar diseases and vice versa, so the topologic information of miRNA/disease similarity network can be used to construct feature factor directly.

For each miRNA, there are 495 similarity scores. We use similarity scores as features to represent each miRNA by a 495-dimensional feature vector. For example, we represent miRNA *m*(*i*) by a feature vector, *SM*(*m*(*i*)) = (*m*_1_, *m*_2_, …, *m*_495_), where *SM*(*m*(*i*)) is the *i*th row vector of *SM* and represents the similarities between *m*(*i*) and all the miRNAs.

For each disease, we can obtain a 383-dimensonal feature vector in a similar way to miRNA, *SD*(*d*(*j*)) = (*d*_1_, *d*_2_, …, *d*_383_), where *SD*(*d*(*j*)) is the *j*th row of matrix *SD*. Therefore, each miRNA-disease pair can be described by an 878-dimensional vector *x* = (*SM*(*m*(*i*)), *SD*(*d*(*j*))). Furthermore, we consider (*SM*(*m*(*i*)), *SD*(*d*(*j*))) as a positive sample if miRNA *m*(*i*) is associated with disease *d*(*j*), otherwise as a negative sample. To construct the balanced dataset, the training set have 5,430 positive samples, and an equal number of samples were randomly selected as negative training examples from the pool of unknown associations. It is possible to use unconfirmed miRNA-disease pairs with association as negative samples, from the perspective of probability, because the miRNA-disease pairs we selected as negative samples account for only 5430 ÷ (495 × 383) ≈ 2.86% of all miRNA-disease pairs, which is negligible [38].

### Hypergraph construction

Firstly, we briefly introduce the hypergraph learning theory. As a generalization of graph, hypergraph represents the structure of data via measuring the similarity between groups of points. Different from a simple graph, an edge in a hypergraph can connect three or more vertices, it can model high-order relations between their vertices by hyperedges, whose influence can be assessed by properly estimating their weights. Obviously, modeling the high-order relationship among objects can improve the predicting performance significantly. Moreover, the quality of the hypergraph structure plays an important role for data modeling. A well constructed hypergraph structure can represent the data correlation accurately, and leading to better performance.

A hypergraph is defined as *G* = (*V, E, w*), where *V* is a set of vertices, *E* is a set of hyperedges and each hyperedge *e* is given a positive weight *w*(*e*). The hypergraph *G* can be denoted by a |*V*| ×|*E*| incidence matrix *H*, in which each entry is defined by

10 $$h(v,e) = \left\{ {\begin{array}{*{20}l} 1 \hfill & {if\, v \in e} \hfill \\ 0 \hfill & { if\, v \notin e} \hfill \\ \end{array} } \right.$$

The degree of vertex *v* ∈ *V* and hyperedge *e* ∈ *E* can be respectively represented as:

11 $$d\left( v \right) \, = \mathop \sum \limits_{e \in E} w\left( e \right)h\left( {v,e} \right)$$

12 $$\delta \left( e \right) \, = \mathop \sum \limits_{{{ }v \in V}} h\left( {v,e} \right)$$

Accordingly, denote *Dv* and *De* as two diagonal matrices of the vertex degrees and the hyperedge degrees, respectively.

Zhou et al. proposed a regularization framework on hypergraph [39], which is defined as

13 $${\text{arg min}}_{f} \{ \lambda R_{{{\text{emp}}}} \left( f \right) + \Omega \left( f \right)\}$$

where *f* is the to-be-learned function, *Ω*(*f*) is a regularizer on the hypergraph, *R*_emp_(*f*) is an empirical loss, and *λ* > 0 is the tradeoff parameter. Usually, the empirical loss *R*_emp_(*f*) is defined as

14 $$R_{{{\text{emp}}}} \left( f \right) = \, ||f - Y||^{{2}}$$

where *Y* is the label matrix of samples. The regularizer on the hypergraph is defined by

15 $$\varOmega \left( f \right) = \frac{1}{2}\mathop \sum \limits_{e \in E} \mathop \sum \limits_{u,v \in V} \frac{w\left( e \right)}{{\delta \left( e \right)}}\left( {\frac{f\left( u \right)}{{\sqrt {d\left( u \right)} }} - \frac{f\left( v \right)}{{\sqrt {d\left( v \right)} }}} \right)$$

Let *Θ* = *D*_*v*_^−(1/2)^*HWD*_e_^−1^*H*^T^*D*_*v*_^−(1/2)^**,** the normalized cost function can be written as

16 $$\varOmega \left( f \right) \, = f^{{\text{T}}} \varDelta f$$

where *Δ* = *I* – *Θ*, which is a positive semi-definite matrix.

**Fig. 2:**
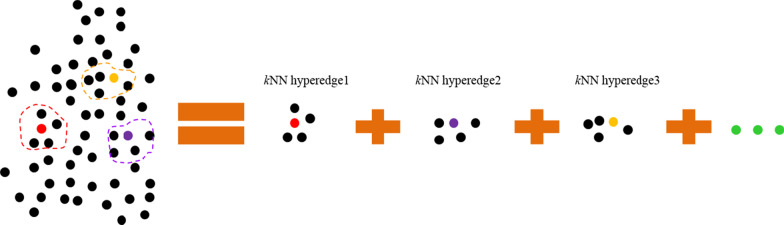
Intuitive illustration of KNN hyperedge generation

In this study, given a set of training samples {*x*_*i*_ |*i* = 1,…, *n*} ∈ ${\mathbb{R}}$^878^, the data matrix *X* = [*x*_1_*,..., x*_*i*_*,..., x*_*n*_]^T^ ∈ ${\mathbb{R}}$^*n*×878^ contains *n* samples in its rows, the corresponding labels matrix *Y* = [*y*_1_*,..., y*_*2*_*,..., y*_*l*_] ∈ ${\mathbb{R}}$^*n*×*l*^, *y*_*i*_ is the label vector of the *i*-th class. A miRNA-disease pairs hypergraph ${\mathcal{G}} = \left( {{\mathcal{V}},{ \mathcal{E}},{ \mathcal{W}}} \right)$ is constructed, and its hyperedge is generated based on the KNN algorithm. Concretely, for each vertex *v*, we search its corresponding *k* nearest neighbors, and use these nearest neighbors to form a hyperedge *e*(*v*). We initialize *k* as 15 here empirically. An illustration on the hyperedge generation process is shown in Fig. 2. Moreover, the diagonal matrix ${\mathcal{W}}$ denote the weights of the hyperedges. All the hyperedges are initialized with an equal weight, *e*.*g*., *w*(*e*) = 1/*n*_*e*_, where *n*_*e*_ is the number of hyperedges.

### Hypergraph learning

The hypergraph learning targets on learning a regularized projection to discriminate different categories. According to Zhang et al. introduction [40], the cost function *F* for learning the projection matrix *P* can be formulated as:

17 $$F = \, \{ \varOmega (P) + \, \lambda R_{emp} (P) + \mu \varPhi (P)\}$$

where *λ* and *μ* are positive parameters, and we empirically set them as 10^1^,10^0^ respectively, which can achieve the best performance. Specifically, hypergraph Laplacian regularizer *Ω*(*P*) is calculated as

18 $$\begin{aligned} \varOmega (P) & = \frac{1}{2}\mathop \sum \limits_{k = 1}^{l} \mathop \sum \limits_{e \in E} \mathop \sum \limits_{u,v \in V} \frac{{W\left( e \right)H\left( {u,e} \right)H\left( {v,e} \right)}}{\delta \left( e \right)}\left( {\frac{{\left( {XP} \right)\left( {u,k} \right)}}{{\sqrt {d\left( u \right)} }} - \frac{{\left( {XP} \right)\left( {v,k} \right)}}{{\sqrt {d\left( v \right)} }}} \right)^{2} \\ & = tr(P^{T} X^{T} \varDelta XP) \\ \end{aligned}$$

where function *tr*(*·*) returns the trace of matrix. The empirical loss term *R*_*emp*_ (*P*) is defined as

19 $$R_{emp} (P) \, = \left| {\left| {XP - Y} \right|} \right|^{{2}}$$

*Φ*(*P*) is a *l*_2_ norm regularizer to avoid over-fitting for *P*, which is defined as:

20 $$\varPhi (P) = \, \left| {\left| P \right|} \right|^{{2}}$$

Consequently, Eq. (17) can be reformed as:

21 $${\text{arg min}}_{P} \left\{ {tr\left( {P^{{\text{T}}} X^{{\text{T}}} \Delta XP} \right) \, + \lambda ||XP - \, Y\left| {\left| {^{{2}} + \mu } \right|} \right|P||^{{2}} } \right\}$$

Such problem is a typical Least Square problem which can be efficiently solved, its solution is as follows:

22 $$P = \lambda (X^{T} \varDelta X + \lambda X^{{\text{T}}} X + \mu I)^{{ - {1}}} X^{{\text{T}}} Y$$

where *I* is an identity matrix. Based on the learned *P*, the relevance score of the unknown miRNA-disease pair *x*^*unk*^ can be obtained by

23 $${\text{S}}\left( {x^{unk} } \right) = x^{unk.} P$$

## Results

### Effect of parameters on the performance of HFHLMDA

**Fig. 3:**
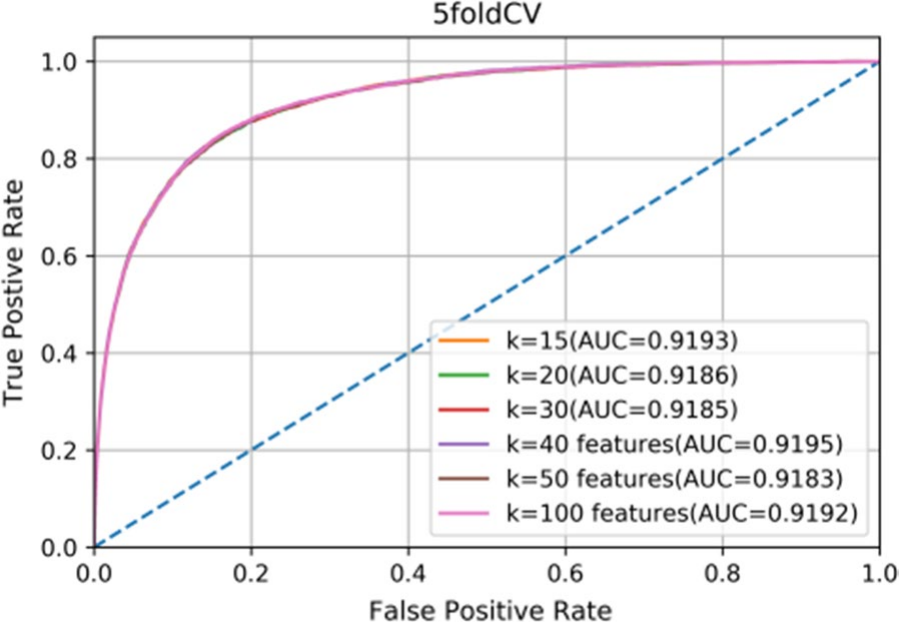
ROC curve and AUC with different values for parameter k

**Fig. 4:**
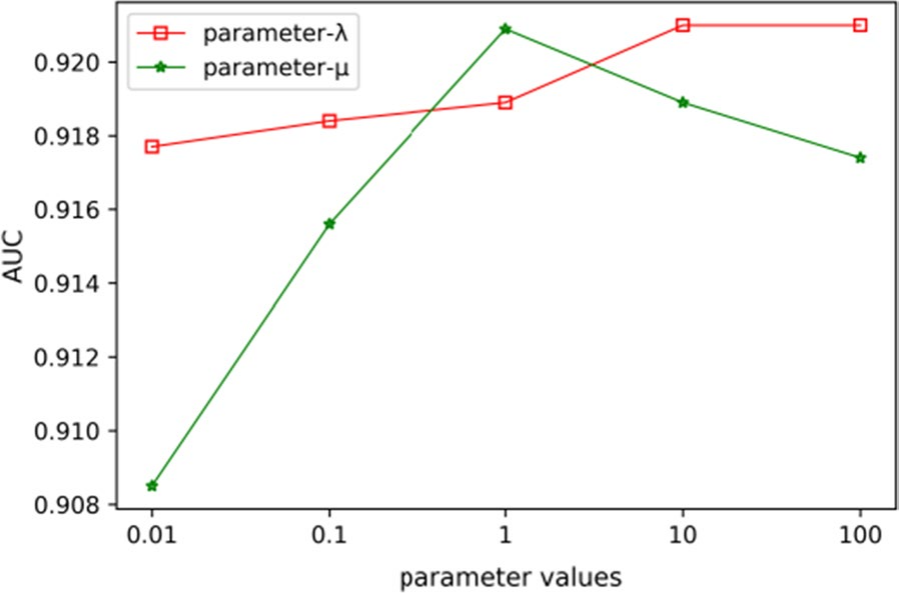
AUC with different values for parameters *λ* and *μ*

In this work, we used KNN algorithm to generate hyperedge, one parameters *k* was included, which represent the number of nearest neighbors of miRNA or disease. In the hypergraph learning section of the Methods, we defined two parameters, namely, *λ* and *μ* to balance the items in Eq. (17), the values of *λ* and *μ* ranged from 10^–2^, 10^–1^, 10^0^, 10^1^ to 10^2^. We conducted a series of experiments on the above parameters to acquire the effects of these parameters. The experimental results are shown in Figs. 3 and 4. In Fig. 3, we can see that regardless of how *k* change, the AUC of fivefold cross validation keep around 0.9187. Thus, for efficiency, we set *k* = 15. Furthermore, Fig. 4 describes the prediction performances of HFHLMDA with different values of *λ* and *μ*. We can see that HFHLMDA obtains the best prediction performance when *λ* is set to be 10^1^ and *μ* is set to be 10^0^.

### Performance evaluation

**Fig. 5:**
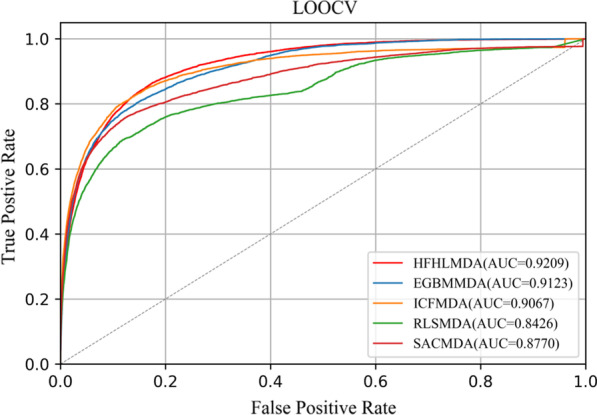
Performance comparisons between HFHLMDA and four classical models in terms of ROC curve and AUC based on LOOCV

Based on the known miRNA–disease associations in HMDDv2.0 database, two validation schemas were used to evaluate the performance of HFHLMDA: LOOCV and fivefold cross validation. We selected four classical computational methods: EGBMMDA [34], ICFMDA [28], RLSMDA [30], and SACMDA [41] to compete with HFHLMDA in cross validation. Specifically, LOOCV selected a known miRNA-disease association in turn as a test sample, and the rest of the associations were considered as training samples. All unknown associations were used as candidate samples. Considering that the Gaussian interaction profile kernel similarity depend on known miRNA-disease associations, the corresponding value of a test sample in matrix A should be set to 0. The predicted score for the test sample was ranked relative to the scores for candidate samples and, each ranking will take turns as a threshold in each fold, if test ranking was above a given threshold, we obtained a successful prediction made by the model. By changing the threshold, we could calculate the corresponding true positive rate (TPR) and false positive rate (FPR). Furthermore, receiver-operating characteristics (ROC) curve could be drawn according to TPR against FPR. The areas under the ROC curve (AUC) was used to evaluate the whole prediction performance. Figure 5 shows the global LOOCV ROC curves for HFHLMDA and other methods. HFHLMDA, EGBMMDA, ICFMDA, RLSMDA and SACMDA obtained AUCs of 0.9209, 0.9123, 0.9067, 0.8426 and 0.8770, respectively. HFHLMDA achieved the better prediction performance.

**Fig. 6:**
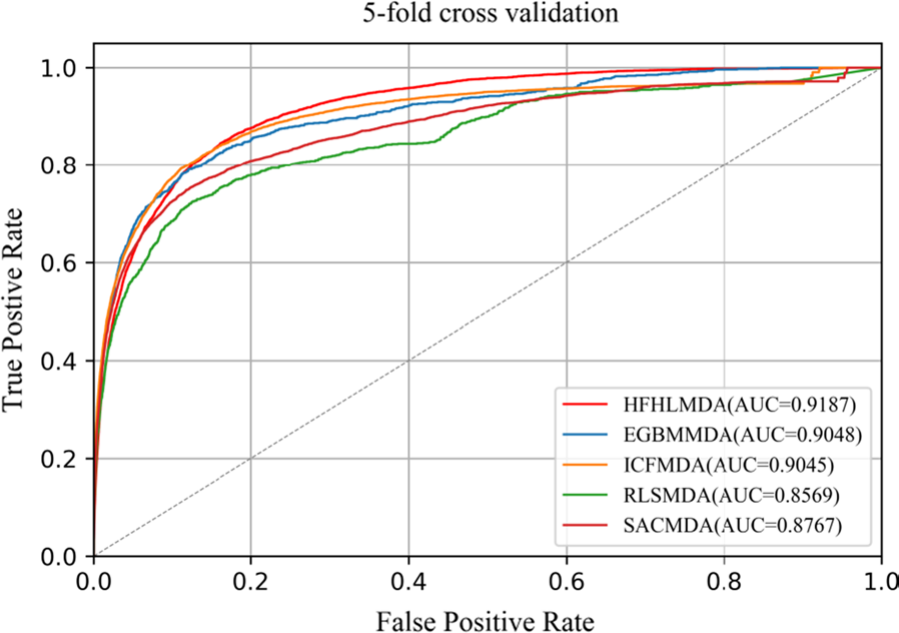
Performance comparisons between HFHLMDA and four classical models in terms of ROC curve and AUC based on fivefold cross validation

As for fivefold cross validation, in order to make the validation more accurate, we repeated fivefold cross validation procedure 100 times. The average AUC values of the five methods (HFHLMDA, EGBMMDA, ICFMDA, RLSMDA, SACMDA) were 0.9187(± 0.0009), 0.9048(± 0.0012), 0.9045(± 0.0008), 0.8569(± 0.0020) and 0.8767(± 0.0011), respectively (see Fig. 6). In summary, under the same dataset, our model outperformed other competitive methods.

### Case studies

Case studies were conducted to further verify the capability of HFHLMDA to predict miRNA-disease associations. We implemented three different kinds of case studies in this study. In the first case study, we conducted HFHLMDA to predict potential disease-miRNA associations taking advantages of known diseases-miRNAs associations included in HMDD v2.0 database. Subsequently, top 50 miRNAs for the investigated disease ranked according to their predicted scores were verified using another two well-known miRNA-disease association databases of dbDEMC [42] and miR2Disease [43]. In the second case study, we simulated the situation where HFHLMDA was conducted for disease without known miRNA associations. More concretely, we removed the known miRNA associations of the disease of interest, after which HFHLMDA was implemented according newly obtained association records. The prediction results were also verified by other databases. The final case study investigated the robustness of HFHLMDA prediction performance. We evaluated the model with a smaller and earlier version HMDDv1.0 database [44].

Esophageal cancer (EC) is one of the most common cancers worldwide, and its 5-year survival rate is about 20% [45]. Study indicate that miR-130b plays an oncogenic role in esophageal squamous cell carcinoma cells by repressing phosphatase and tensin homolog expression and Akt phosphorylation [46]. Therefore, specific and sensitive biomarkers for diagnosis and targeted therapy of EC are urgently needed. As the first type of case study, 10 out of top 10, 28 out of top 30, 45 out of top 50 predicted esophageal neoplasms related miRNAs were confirmed by dbDEMC (See Table 1).

**Table 1:** The top 50 predicted miRNAs associated with esophageal cancer

miRNA	Evidence	miRNA	Evidence
hsa-mir-221	dbDEMC	hsa-mir-9	dbDEMC
hsa-mir-125b	dbDEMC	hsa-mir-24	dbDEMC
hsa-mir-29a	dbDEMC	hsa-mir-132	dbDEMC
hsa-mir-206	dbDEMC	hsa-mir-224	dbDEMC
hsa-mir-17	dbDEMC	hsa-mir-23a	dbDEMC
hsa-mir-16	dbDEMC	hsa-let-7d	dbDEMC
hsa-mir-29b	dbDEMC	hsa-mir-195	dbDEMC
hsa-mir-222	dbDEMC	hsa-mir-335	dbDEMC
hsa-mir-1	dbDEMC	hsa-mir-124	dbDEMC
hsa-mir-146b	dbDEMC	hsa-mir-93	dbDEMC
hsa-mir-182	dbDEMC	hsa-mir-106a	dbDEMC
hsa-mir-122	Unconfirmed	hsa-mir-140	dbDEMC
hsa-mir-181a	dbDEMC	hsa-mir-30a	dbDEMC
hsa-mir-18a	dbDEMC	hsa-mir-184	Unconfirmed
hsa-mir-106b	dbDEMC	hsa-mir-429	dbDEMC
hsa-let-7e	dbDEMC	hsa-let-7i	dbDEMC
hsa-mir-200b	dbDEMC	hsa-let-7f	Unconfirmed
hsa-mir-20b	dbDEMC	hsa-mir-134	dbDEMC
hsa-mir-19b	dbDEMC	hsa-mir-27b	dbDEMC
hsa-mir-133b	dbDEMC	hsa-mir-23b	dbDEMC
hsa-let-7 g	dbDEMC	hsa-mir-152	dbDEMC
hsa-mir-181b	dbDEMC	hsa-mir-96	dbDEMC
hsa-mir-15b	dbDEMC	hsa-mir-193b	dbDEMC
hsa-mir-103a	Unconfirmed	hsa-mir-138	Unconfirmed
hsa-mir-142	dbDEMC	hsa-mir-125a	dbDEMC

The first column records top 1–25 related miRNAs. The third column records the top 26–50 related miRNAs

Hepatocellular carcinoma (HC) is a complex polygenetic disease ascribed to the interactions between genetic predisposition and environmental factors [47]. The discovery of vital target for genetic therapy are of great clinical significance to the improvement of the comprehensive effect of HC. For example, miR-122, let-7 family, and miR-101 are down-regulated in HC, suggesting that it is a potential tumor suppressor of HC. miR-221 and miR-222 are up-regulated in HCC and may act as oncogenic miRNAs in hepatocarcinogenesis [48]. We took hepatocellular carcinoma as the second kind of case study. Finally, 49 out of top 50 miRNAs were experimentally confirmed by HMDD v2.0, dbDEMC and miR2Disease (See Table 2).

**Table 2:** The top 50 predicted miRNAs associated with hepatocellular carcinoma

miRNA	Evidence	miRNA	Evidence
hsa-mir-21	HMDD; miR2disease	hsa-let-7b	HMDD; miR2disease
hsa-mir-155	HMDD; miR2disease; dbDEMC	hsa-mir-122	HMDD; miR2disease; dbDEMC
hsa-mir-146a	HMDD; miR2disease; dbDEMC	hsa-mir-18a	HMDD; miR2disease; dbDEMC
hsa-mir-221	HMDD; miR2disease; dbDEMC	hsa-mir-106b	HMDD; miR2disease; dbDEMC
hsa-mir-125b	HMDD; miR2disease	hsa-mir-200a	HMDD; miR2disease; dbDEMC
hsa-mir-145	HMDD; miR2disease; dbDEMC	hsa-mir-223	HMDD; miR2disease
hsa-mir-29a	HMDD; dbDEMC	hsa-mir-150	HMDD; miR2disease; dbDEMC
hsa-mir-206	Unconfirmed	hsa-mir-19b	HMDD; miR2disease
hsa-mir-17	HMDD; miR2disease	hsa-mir-29c	HMDD; dbDEMC
hsa-mir-16	HMDD; miR2disease; dbDEMC	hsa-mir-143	miR2disease; dbDEMC
hsa-mir-29b	HMDD; dbDEMC	hsa-let-7g	HMDD; miR2disease
hsa-mir-199a	HMDD; miR2disease; dbDEMC	hsa-mir-200b	HMDD; miR2disease
hsa-mir-214	HMDD; miR2disease; dbDEMC	hsa-mir-210	HMDD; dbDEMC
hsa-mir-20a	HMDD; miR2disease; dbDEMC	hsa-mir-126	HMDD; miR2disease; dbDEMC
hsa-mir-92a	HMDD; miR2disease	hsa-mir-20b	HMDD; dbDEMC
hsa-mir-222	HMDD; miR2disease; dbDEMC	hsa-let-7c	HMDD; miR2disease; dbDEMC
hsa-mir-1	HMDD; miR2disease	hsa-mir-34c	HMDD
hsa-mir-34a	HMDD; miR2disease; dbDEMC	hsa-mir-141	HMDD; miR2disease
hsa-mir-15a	HMDD; miR2disease; dbDEMC	hsa-mir-133b	HMDD
hsa-mir-182	HMDD; miR2disease	hsa-mir-224	HMDD; miR2disease; dbDEMC
hsa-mir-146b	HMDD	hsa-mir-15b	HMDD; dbDEMC
hsa-mir-181a	HMDD; miR2disease; dbDEMC	hsa-mir-133a	miR2disease
hsa-mir-499a	HMDD	hsa-mir-181b	HMDD; miR2disease; dbDEMC
hsa-let-7e	HMDD; miR2disease; dbDEMC	hsa-mir-200c	HMDD
hsa-let-7a	HMDD; miR2disease; dbDEMC	hsa-mir-19a	HMDD; miR2disease; dbDEMC

The first column records top 1–25 related miRNAs. The third column records the top 26–50 related miRNAs

Breast Neoplasms is the most common malignancy in women, accounting more than 40,000 deaths each year [49]. Data have shown that the number of affected people is climbing, and a forecast deemed that there will be nearly 3.2 million new patients per year by 2050 [50]. In breast cancer, approximately one-fifth of metastatic patients survive 5 years [51]. Researchers have found that many miRNAs are associated with breast neoplasms by clinical experiments, such as mir‐155 and mir‐21, both of which can lead to Breast Neoplasms tumorigenesis or metastasis [52]. We took breast neoplasms as the last kind of case study, in which we got the prediction with HFHLMDA using HMDDv1.0 database. Then, we verified the predicted potential breast neoplasms related miRNAs in other databases. At last, 48 out of top 50 miRNAs were experimentally confirmed by HMDD v2.0, dbDEMC and miR2Disease (See Table 3).

**Table 3:** The top 50 predicted miRNAs associated with breast neoplasms

miRNA	Evidence	miRNA	Evidence
hsa-mir-16	HMDD; dbDEMC	hsa-mir-148a	HMDD; miR2Disease; dbDEMC
hsa-mir-150	dbDEMC	hsa-mir-101	HMDD; miR2Disease; dbDEMC
hsa-mir-96	HMDD; miR2Disease; dbDEMC	hsa-mir-29c	HMDD; miR2Disease; dbDEMC
hsa-mir-223	HMDD; dbDEMC	hsa-mir-342	HMDD; miR2Disease; dbDEMC
hsa-mir-92a	HMDD	hsa-mir-372	dbDEMC
hsa-let-7 g	HMDD; dbDEMC	hsa-mir-30e	Unconfirmed
hsa-let-7e	HMDD; dbDEMC	hsa-mir-92b	dbDEMC
hsa-let-7i	HMDD; miR2Disease; dbDEMC	hsa-mir-193b	HMDD; miR2Disease; dbDEMC
hsa-mir-373	HMDD; miR2Disease; dbDEMC	hsa-mir-27a	HMDD; miR2Disease; dbDEMC
hsa-let-7b	HMDD; dbDEMC	hsa-mir-130a	dbDEMC
hsa-mir-199b	HMDD; dbDEMC	hsa-mir-195	HMDD; miR2Disease; dbDEMC
hsa-mir-183	HMDD; dbDEMC	hsa-mir-184	dbDEMC
hsa-mir-106a	dbDEMC	hsa-mir-126	HMDD; miR2Disease; dbDEMC
hsa-mir-23b	HMDD; dbDEMC	hsa-mir-152	HMDD; miR2Disease; dbDEMC
hsa-let-7c	HMDD; dbDEMC	hsa-mir-196b	dbDEMC
hsa-mir-32	dbDEMC	hsa-mir-660	dbDEMC
hsa-mir-15b	dbDEMC	hsa-mir-454	Unconfirmed
hsa-mir-191	HMDD; miR2Disease; dbDEMC	hsa-mir-224	HMDD; dbDEMC
hsa-mir-212	dbDEMC	hsa-mir-26a	HMDD; miR2Disease; dbDEMC
hsa-mir-24	HMDD; dbDEMC	hsa-mir-26b	HMDD; dbDEMC
hsa-mir-99b	dbDEMC	hsa-mir-203	HMDD; miR2Disease; dbDEMC
hsa-mir-181a	HMDD; miR2Disease; dbDEMC	hsa-mir-198	dbDEMC
hsa-mir-137	HMDD; dbDEMC	hsa-mir-142	HMDD; dbDEMC
hsa-mir-31	HMDD; miR2Disease; dbDEMC	hsa-mir-208b	HMDD; miR2Disease; dbDEMC
hsa-mir-32	dbDEMC	hsa-mir-95	HMDD; dbDEMC

The first column records top 1–25 related miRNAs. The third column records the top 26–50 related miRNAs

The aforementioned case studies indicate that HFHLMDA has good prediction performance. HFHLMDA can efficiently predict disease-related miRNAs based on known miRNA-disease associations, disease semantic similarity and miRNA functional similarity, and a disease without known associations also can be predicted.

## Discussion

In this work, we developed a new computational model based on hypergraph learning to predict potential miRNA‐disease associations. Several important factors contribute to the excellent performance of our model. First, high-dimensionality features. Based on a credible assumption that functionally similar miRNAs tend to have associations with phenotypically similar diseases. We use the miRNAs or diseases similarity scores directly as a feature factor, with a dimension of up to 878, which contains all similar information about miRNAs or diseases. Second, hypergraph is suitable to represent local group information and the high-order relationship of data, and can completely represent the complex relationships among miRNA-disease pairs. Different from the simple-graph learning methods consider only the pair-wise relationship between two samples, and they ignore the relationship in a higher-order, hypergraph learning aims to get the relationship between several samples in a higher order. Hypergraph learning is a kind of graph clustering algorithm, the process of graph clustering is actually the optimization of graph partition. The purpose of optimization is to reduce the similarity between sub-graphs and increase the similarity within sub-graphs. Hypergraph-based models have proven to be beneficial for a variety of classification/clustering tasks, and we think it can also be applied to different fields of bioinformatics, such as drug-disease associations [53], miRNA–drug interactions [54].

Despite the practicability and efficiency of HFHLMDA, there still has some limitations. Since our method is based on machine learning techniques, negative samples are required during the training process. However, experimentally confirmed negative samples are difficult to obtain. To resolve this issue, we have randomly selected a subset of unknown miRNA–disease associations as negative instances. In addition, in our method, after the hypergraph has been constructed, it never changes during the learning process, leading to a static hypergraph structure learning mechanism. However, it is uneasy to guarantee that the generated hypergraph structure is optimal and suitable for all applications. In future work, it is necessary to investigate the hypergraph structure optimization, leading to a dynamic hypergraph structure learning scheme.

## Conclusion

Increasing evidence indicates that aberrant expression of miRNAs is closely related to the occurrence and development of human complex diseases. Understanding the underlying mechanisms of miRNAs in diseases is becoming an urgent problem worldwide. Compared with traditional methods, the computational model developed for processing heterogeneous biological big data is more efficient and convenient. To predict potentially disease-related miRNAs, we proposed a hypergraph learning method called HFHLMDA. Both cross-validation and case studies had proved the effectiveness of HFHLMDA in predicting potential miRNA-disease associations.

## Data Availability

The datasets used during this study is provided by Li et al. [35]. Please download the data from http://www.cuilab.cn/hmdd/ or contact the authors for data requests.

## Abbreviations

KNN: K-Nearest-Neighbor
HMDD: Human microRNA disease database
dbDEMC: Database of differentially expressed miRNAs in human cancers
ROC: Receiver operating characteristics
AUC: The area under the ROC curve
LOOCV: Leave-one-out cross validation
